# Quantitative application of dual-phase 99mTc-sestamibi SPECT/CT imaging of parathyroid lesions: identification of optimal timing in secondary hyperparathyroidism

**DOI:** 10.1186/s40658-023-00548-5

**Published:** 2023-04-20

**Authors:** Yuhua Wang, Ye Liu, Na Li, Kang Xu, Wanchun Zhang

**Affiliations:** 1 Department of Nuclear Medicine, Shanxi Bethune Hospital, Shanxi Academy of Medical Sciences, Tongji Shanxi Hospital, Third Hospital of Shanxi Medical University, Taiyuan, 030032 China; 2 Tongji Hospital, Tongji Medical College, Huazhong University of Science and Technology, Wuhan, 430030 China

**Keywords:** Hyperparathyroidism, 99mTc-MIBI, SPECT/CT, Maximum standardized uptake, Chronic kidney disease

## Abstract

**Purpose:**

In this retrospective study, we compared the maximum standardized uptake values (SUVmax) of parathyroid lesions and the target-to-background ratio (TBR) of parathyroid lesions to thyroid tissue in early-phase single-photon emission computed tomography/computed tomography (SPECT/CT) versus delayed-phase SPECT/CT in patients with secondary hyperparathyroidism (SHPT) in order to determine the optimal timing of 99mTc- methoxyisobutylisonitrile (99mTc-MIBI) SPECT/CT imaging.

**Methods:**

Seventeen patients with a history of chronic kidney failure stage 5 on hemodialysis, underwent pre-operative parathyroid scintigraphy for detection and localization of parathyroid lesions. Retrospective analysis was conducted for lesions with focal accumulation of 99mTc-MIBI. All patients underwent dual-phase 99mTc-MIBI parathyroid scintigraphy and dual-phase SPECT/CT. SUVmax of parathyroid lesions and thyroid tissues was measured.

**Results:**

Mean SUVmax of parathyroid lesions was 4.86 on early-phase and 2.58 on delayed-phase SPECT/CT, respectively. Mean TBR was 1.14 on early phase and 1.48 on delayed-phase SPECT/CT, respectively. Statistically significant differences in SUVmax and TBR between dual-phase SPECT/CT were observed (*P* < 0.001).

**Conclusions:**

Delayed-phase SPECT/CT in SHPT is required because of the better image contrast.

## Background

Secondary hyperparathyroidism (SHPT) is one of the most common serious complications in patients with chronic renal failure on long-term hemodialysis. It is characterized by increased parathyroid hormone (PTH) secretion and parathyroid cell proliferation leading to a metabolic disorder (altered calcitriol, calcium and phosphorus) and enlargement of the parathyroid glands. Elevated PTH is considered both a consequence and cause of the metabolic bone disorder, and it has been linked with chronic kidney disease (CKD) osteodystrophy, where high bone turnover leads to higher risk of fractures, hyperphosphatemia, vascular and tissue calcification, anemia hyporesponsive to erythropoietin therapy, worse health-related quality of life, and increased mortality [1]. Treatment using surgical parathyroidectomy (sPTX) is common in cases in which PTH control is insufficient with a Vitamin D receptor agonist (VDRA) and a calcimimetic [2]. sPTX is required in about 15% of patients after 10 years and in 38% after 20 years of ongoing dialysis therapy [3] and has been shown to significantly decrease all-cause mortality in end-stage kidney disease patients with SHPT [4, 5]. Most patients on dialysis with SHPT have multiple enlarged parathyroid glands and frequent variations in parathyroid anatomical location [1]. Meta-analysis showed that 15.9% of 7529 parathyroid glands were present in an ectopic location with 11.6% in the neck and 4.3% in the mediastinum [6]. Another study of 177 patients showed that 22% had supernumerary parathyroid glands (number of parathyroid glands ≥ 5) and 36.7% had ectopic parathyroid glands [7]. The thymus is the most common location for ectopic parathyroid glands [8]. Therefore, the accurate preoperative detection and localization of hyperplastic parathyroid glands are critical for a successful surgery.

Radionuclide imaging with scintigraphy, performed with a large field-of-view gamma camera, is most commonly used for anatomical localization of parathyroid tissue, particularly in the case of ectopic glands. 99mTc-methoxyisobutylisonitrile (99mTc-MIBI) dual-phase scanning is employed in many nuclear medicine departments exploiting the phenomenon whereby hyperfunctional parathyroid tissue retains radiotracer for prolonged periods compared to normal thyroid tissue. With the development of single-photon emission computed tomography/computed tomography (SPECT/CT), the combination of SPECT/CT and 99mTc-MIBI dual-phase imaging technology has become a common technique for accurate localization [9]. 99mTc-MIBI SPECT/CT was shown to accurately localize 80% parathyroid adenomas according to the Perrier classification [10], its diagnostic accuracy was significantly improved compared to planar imaging or other technologies [5, 10–12]. The EANM practice guideline also recommended performing at least one SPECT/CT study with 99mTc-MIBI [13]. Most reports advised performing SPECT/CT in the early phase [14], while a few advised using the delayed phase [15]. There are also studies showing that dual-phase SPECT/CT had higher sensitivity than single-phase SPECT/CT alone [16, 17]. The minimally invasive surgery rate after multi-phase SPECT/CT was up to 100% [18]. Therefore, controversy persists regarding optimal timing of SPECT/CT. As far as we understand, most published research related to primary hyperparathyroidism (PHPT) and visual judgment has been employed for qualitative analysis of SPECT/CT. With technical improvements in reconstruction algorithms for SPECT/CT, the SUV is now commonly used as a powerful tool for measuring tissue uptake, and it has been used in SPECT/CT [19–21]. The aim of our study was to employ quantitative measurement techniques to assess the degree of 99mTc-MIBI concentration in parathyroid lesions comparing the early phase and delayed phase to determine the optimal timing of 99mTc-MIBI SPECT/CT imaging in SHPT patients.

## Methods

### Patients

We performed a retrospective study of 17 consecutive patients with CKD stage 5 who underwent parathyroidectomy for SHPT at our institute between October 2021 and August 2022. The study was approved by the Ethics Review Board of Our Hospital (approval number: YXLL-2022-107). All the patients were on hemodialysis. Biochemical indices, such as serum calcium (Ca), phosphorus (P), alkaline phosphatase (ALP), parathyroid hormone (PTH), urea nitrogen (BUN) and creatinine (Scr) levels, were measured before surgery. All patients underwent dual-phase planar imaging with 99mTc- MIBI and SPECT/CT imaging on a Siemens scanner.

### 99mTc-sestamibi scintigraphy and 99mTc-sestamibi SPECT/CT acquisition

Imaging acquisition was performed on a double-head gamma-camera equipped with low-energy high-resolution (LEHR) collimators (Siemens Symbia Intevo Bold). After injection of 555 MBq (15 mCi) of 99mTc- MIBI, early and delayed parathyroid scans were obtained 15 min (min) and 2 h (h) after the injection, respectively. Anterior neck images were obtained in a 256 × 256 matrix, gathering 500 k counts per position, with a 20% energy window centered on the 140 keV photopeak after 15 min and 2 h. SPECT/CT imaging was performed immediately after both the early and delayed planar images. During the SPECT study, 30 stops were acquired, with 20 s acquisition time per frame and a matrix of 256 × 256 pixels. CT images were acquired with the tube voltage set at 100 kV, tube current determined by automatic dose modulation with a set reference current of 60 mAs.

### Qualitative and quantitative analysis of 99mTc-MIBI dual-phase SPECT-CT

Reconstructed images were evaluated visually and quantitatively by two experienced nuclear medicine physicians blinded both to surgical findings and pathological results. Positive 99mTc-MIBI SPECT/CT scans indicated a fixed concentration in neck or mediastinum on SPECT imaging, with a parenchymal space-occupying lesion (independent soft tissue mass) in the corresponding position on CT imaging.

For quantitative analysis of dual-phase and SPECT/CT imaging, we used a nuclear medicine software package. The voxel-based volume activities obtained were converted by use of the xSPECT Quant application to SUVs by accounting for the patient’s weight, the injected activity, the residual activity in the syringe after administration and the time between injection and acquisition. Three-dimensional volumes of interest were placed over the positive lesion and over the bilateral thyroid gland tissue. SUVmax values of the lesions and thyroid were recorded in the study. The target-to-background ratio (TBR) was calculated using the SUVmax of the parathyroid lesion and the ipsilateral thyroid lobe. Retention indices (RI) for the parathyroid lesion (RI-P) and the thyroid gland (RI-T), related to early-phase scanning, were calculated according to equation [19]: RI = (SUVmax_delayed_ − SUVmax_early_)/SUVmax_early._

### Statistical analysis

Statistical analyses were performed using R software version 4.0.3 (R Core Team, 2014). Variables were expressed as mean ± SD, or median with interquartile range, and categorical variables were summarized as counts or percentages. Rank-sum tests for paired samples were used for dual-phase parathyroid lesion SUVmax and TBR. Fisher's exact probability method was used for comparing categorical data. Multiple linear regression was carried out to analyze the factors affecting TBR. Correlation heat maps were drawn with R software. Statistical significance was set at *P* less than 0.05.

## Results

### Patients

In all 17 patients included in the study, the primary diagnosis was stage 5 CKD. All the patients were on hemodialysis, and the mean duration of dialysis was 10 years. There were 13 males and four females, with a mean age of 37 (range, 35–48) years. All patients had significantly increased serum PTH preoperatively, which decreased postoperatively. Table 1 shows the baseline characteristics for the 17 patients in the study. Among the lesions resected, 36 were pathologically confirmed as parathyroid hyperplasia and nine were parathyroid adenomas. Of the 45 confirmed lesions, 26 were on the dorsal side of the thyroid lobe, 17 were in the inferior pole of the thyroid lobe, and two were in the upper mediastinum.

**Table 1:** Baseline characteristics of patients included in the study

Indices (unit)	Normal range		*N*
Sex, *n* (%)			17
Male		13 (76.5%)	
Female		4 (23.5%)	
Age (years)		37 [35; 48]	17
PTH (pg/ml)	12–88	1656 [1170; 2801]	17
P (mmol/L)	0.85–1.51	2.00 [1.51; 2.46]	17
Ca (mmol/L)	2.11–2.52	2.50 [2.44; 2.55]	17
ALP (IU/L)	50–135	277 [117; 765]	17
BUN (mmol/L)	3.1–8.8	17.2 [14.5; 28.5]	17
SCr (umol/L)	58–96	903 [628; 1034]	17
Dialysis duration (years)		10.0 [5.00; 12.0]	17

Data are represented as median [interquartile range]

*PTH* parathyroid hormone, *P* phosphorus, *Ca* calcium, *ALP* alkaline phosphatase, *BUN* blood urea nitrogen, *Scr* creatinine

### Image findings and quantification

**Fig. 1:**
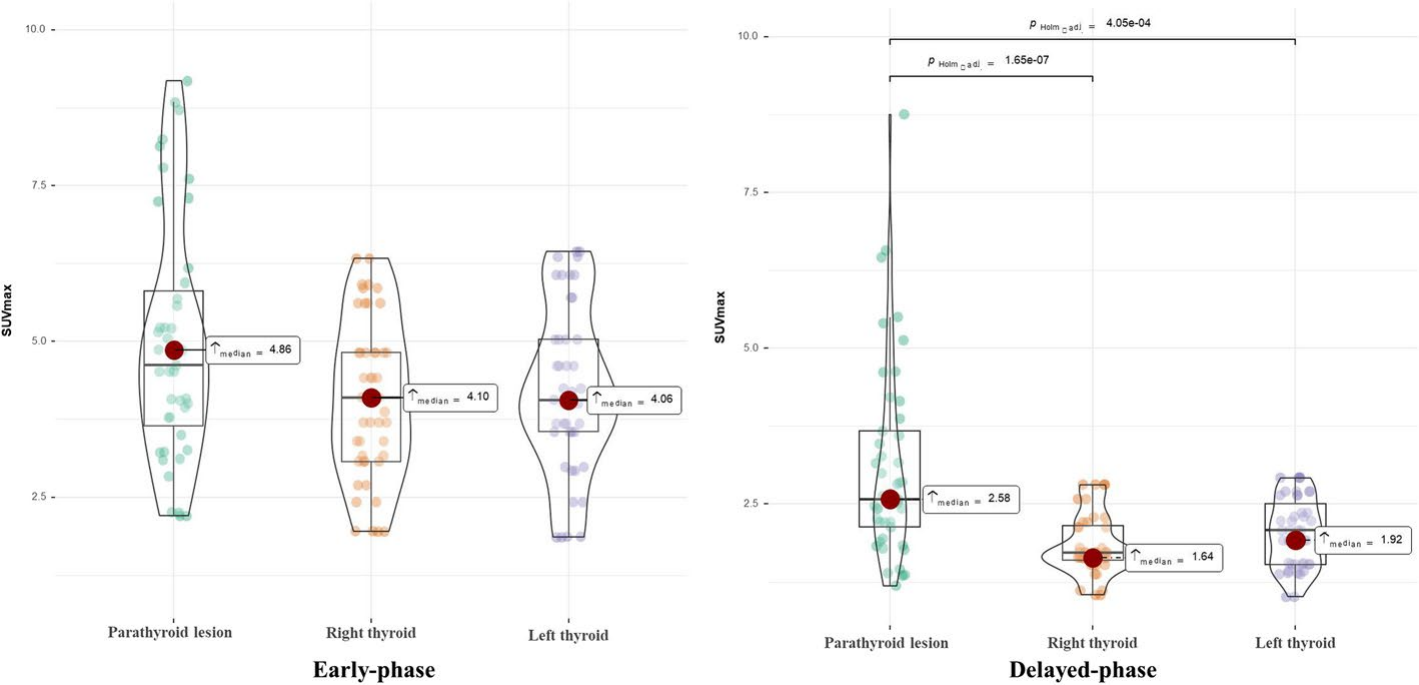
SUVmax values of parathyroid lesions and both thyroid lobes in early-phase and in delayed-phase images. Median SUVmax of parathyroid lesions in early phase was 4.86, which was higher than both thyroid lobes without significant differences. Median SUVmax of parathyroid lesions in delayed phase was 2.58, which was higher than in both thyroid lobes, the differences being statistically significant (both *P* < 0.001)

**Fig. 2:**
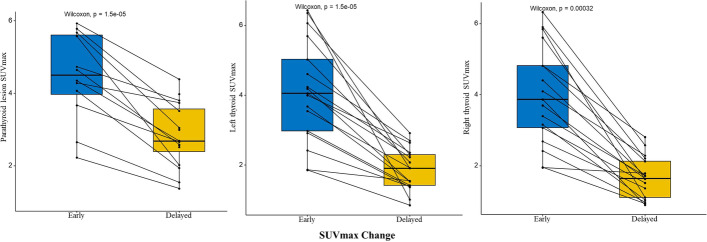
SUVmax changes in early and delayed phase in parathyroid lesions and both thyroid lobes. Boxplots indicate median value (black line), first and third quartile ranges (box). The SUVmax of both parathyroid lesions (left) and thyroid dual lobes (middle, thyroid left lobe; right, thyroid right lobe) in early phase was higher than in delayed phase with significant differences. Rank-sum testing for paired samples used for the difference between two phases in parathyroid lesions and thyroid dual lobes. All the differences were statistically significant (*P* < 0.001)

**Fig. 3:**
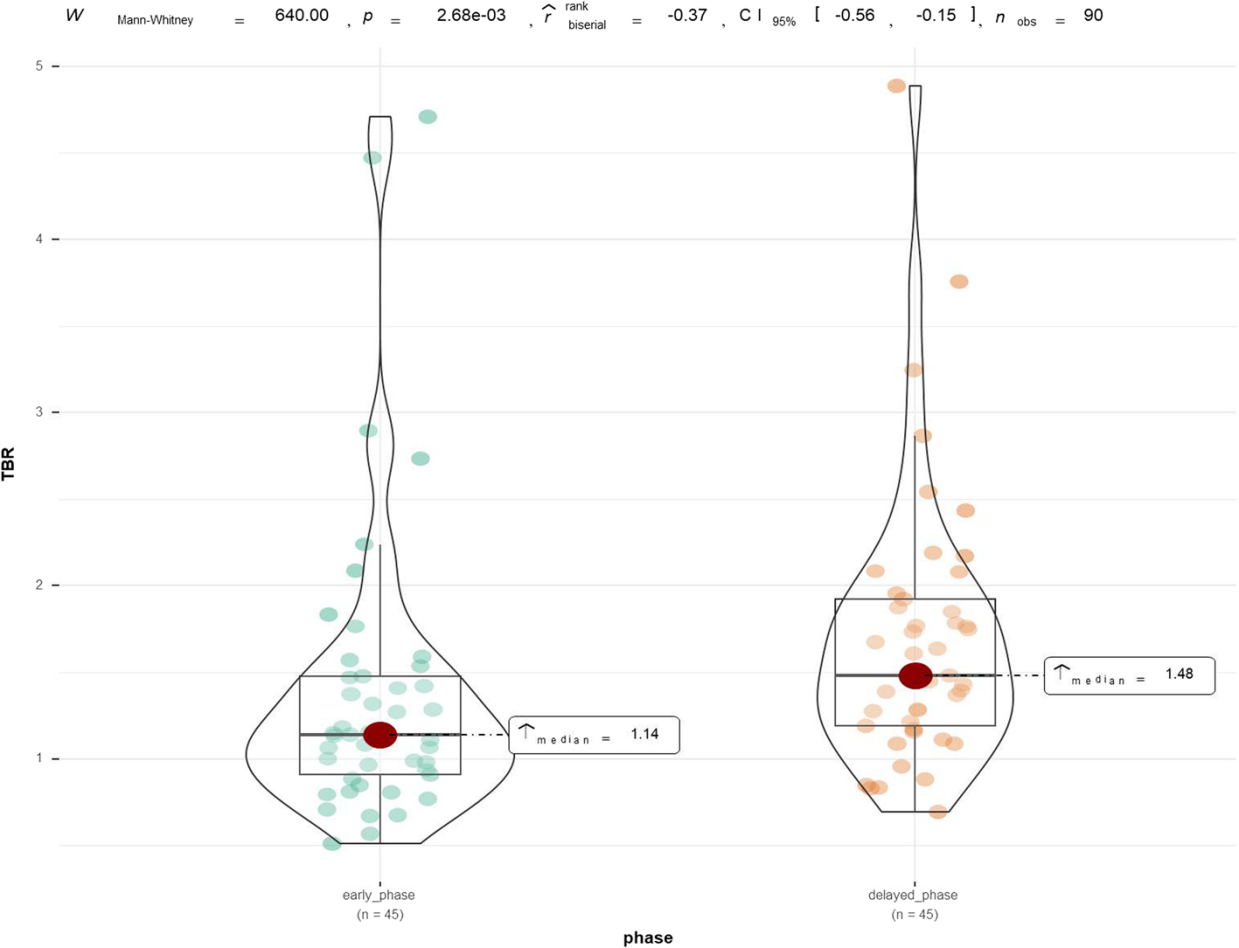
Violin plot analysis comparing the TBR of parathyroid lesions in early-phase and delayed-phase images. The green group represents the early phase, whereas the orange represents the delayed phase. The red oval indicates the median value, while the size of the black box indicates the first and third quartile ranges. The rank-sum test of independent samples was used, and the TBR level in the delayed phase was significantly higher than in the early phase (*P* < 0.001). *TBR* is the tumor-to-background counts ratio

Forty-five lesions of focally increased accumulation of 99mTc-MIBI, with matching masses on the SPECT/CT, were identified. Mean SUVmax values from parathyroid lesions, left thyroid lobes and right thyroid lobes are shown in Fig. 1*.* Median early-phase SUVmax of parathyroid lesions was 4.86 compared with 4.10 in the right lobe and 4.06 in the left lobe of the thyroid with no statistical differences. Delayed-phase SUVmax of parathyroid lesions was 2.58, which was significantly higher than 1.64 in the right lobe and 1.92 in the left lobe of the thyroid. The SUVmax values of left and right thyroid lobes did not differ significantly. The changes in SUVmax of parathyroid lesions and thyroid lobes between early- and delayed-phase images are shown in Fig. 2. Pair-wise testing showed that the delayed-phase SUVmax of parathyroid lesions and thyroid tissue was significantly lower than in the early phase (*P* < 0.001). Analyzed by lesion number, median TBR on delayed-phase imaging was 1.48 which was significantly higher than 1.14 in the early phase (*P* < 0.001) (Fig. 3).

**Fig. 4:**
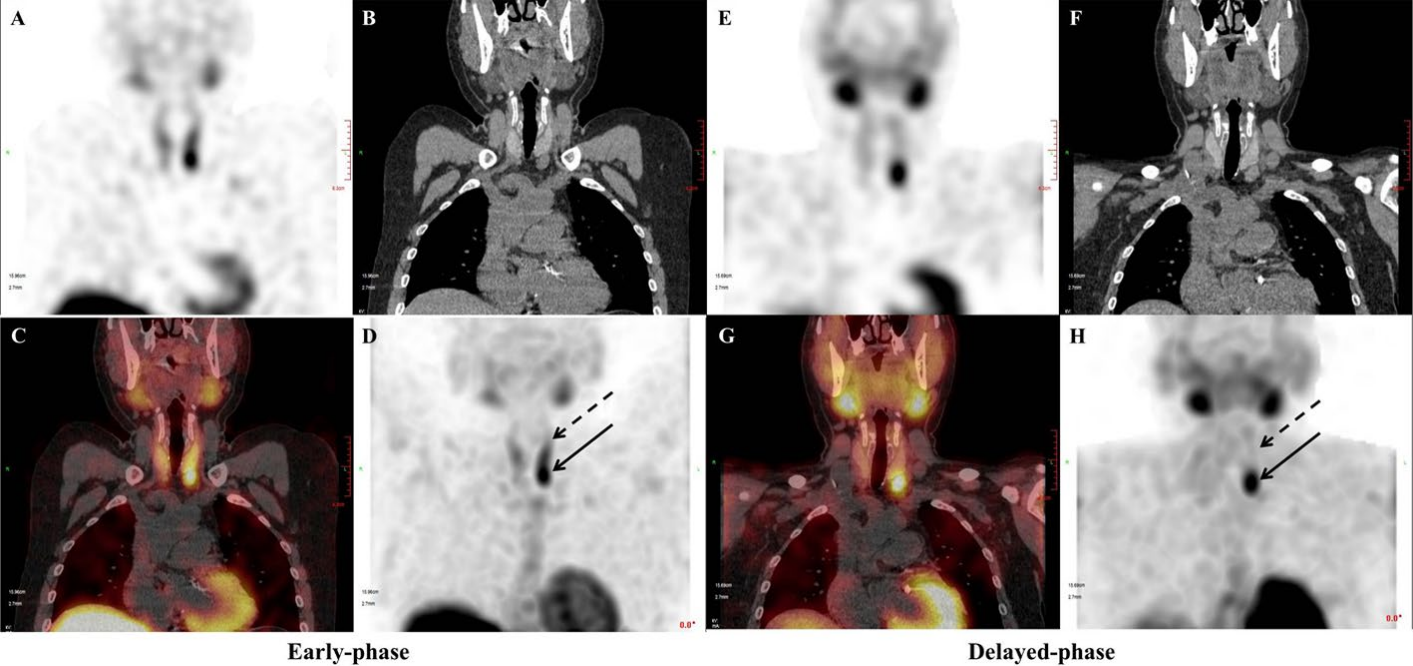
Early phase of coronal SPECT (**A**), coronal CT (**B**), coronal SPECT/CT (**C**) and MIP (**D**) images from 99mTc-MIBI displaying a clear thyroid image (dotted arrow), with focal imaging agent concentration below the inferior pole of the left lobe (arrow). Delayed phase of coronal SPECT (**E**), coronal CT (**F**), coronal SPECT/CT (**G**) and MIP (**H**) images showed significant tracer washout from the thyroid (dotted arrow) while high concentration persisted in the lesion below the inferior pole of its left lobe (arrow). *MIP* is maximum intensity projection

RI values differed between parathyroid lesions and thyroid gland. Mean RI-P, right RI-T and left RI-T were − 41% ± 19%, − 56% ± 15% and − 52% ± 15%, respectively. We found a significant difference between RI-P and right RI-T, and between RI-P and left RI-L (both *P* < 0.05), indicating that the washout of ^99m^Tc-MIBI was slower from parathyroid lesions than from the thyroid lobes (Fig. 4).

### Correlation of TBR with serum biochemistry

**Fig. 5:**
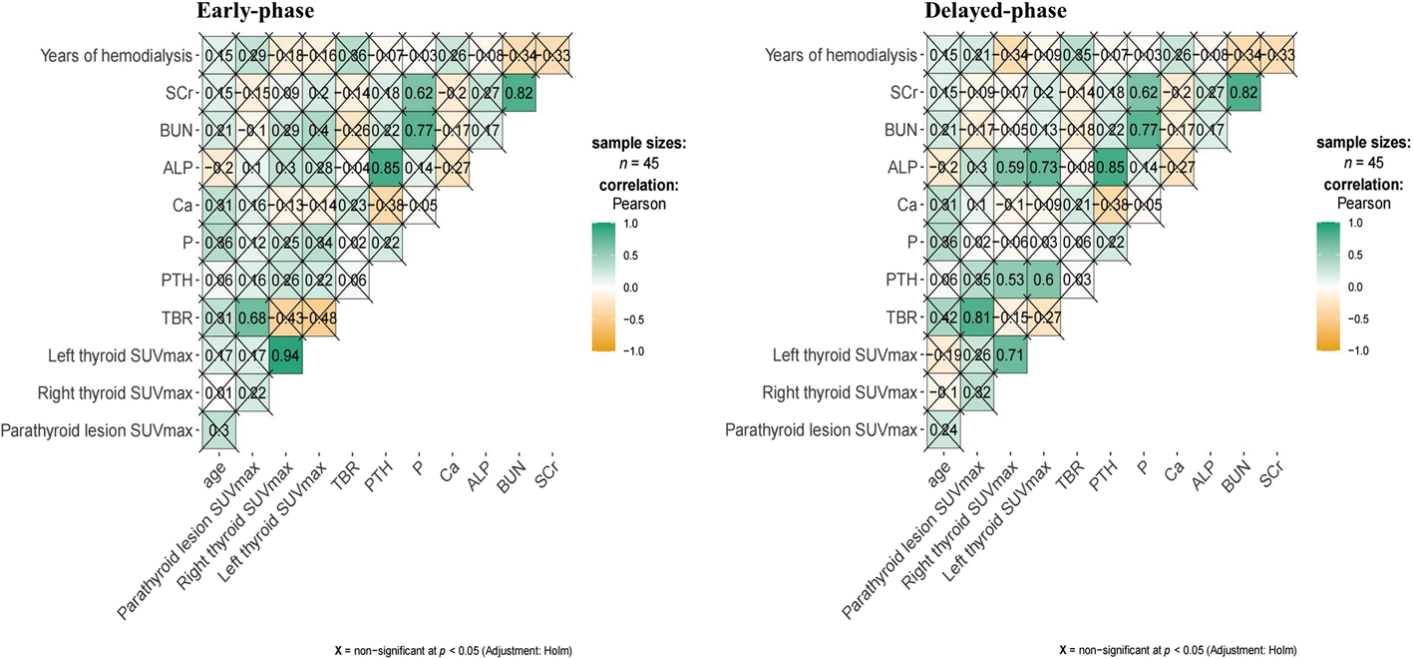
Correlations of TBR and SUVmax with serum biochemistry. The only factor significantly associated with TBR was parathyroid lesion SUVmax. The correlation tests between other factors and TBR showed no statistical significance

Multiple linear regression demonstrated that age, years of hemodialysis and serum PTH, P, Ca, ALP, BUN, SCr did not significantly correlate with parathyroid lesion SUVmax (Fig. 5). There were also no statistically significant correlations between TBR and serum biochemistry.

## Discussion

99mTc-MIBI is a liposoluble, intracellular and cationic tracer that accumulates in abnormal parathyroid gland tissue, particularly if rich in oxyphil cells which contain abundant mitochondria [22]. 99mTc-MIBI scintigraphy is used most commonly to localize parathyroid lesions based on this mechanism [6, 23]. Previous studies have reported that the value of 99mTc-MIBI imaging in SHPT is less than in PHPT [24]; one possible cause is relatively low uptake [24, 25]. The SUVmax levels of parathyroid lesions in our study on early-phase and delayed-phase scanning are both lower than reported in PHPT [19, 21] as has been demonstrated, in vitro, by radiographic monitoring [26]. The factors that influence 99mTc-MIBI accumulation in the parathyroid glands include glandular weight, pathological volume, regional blood flow, oxyphil cell, mitochondrial content, etc. [24, 27–29]. The study [30] by Suh also showed that hyperplasia had a lower SUV than adenomas. Most parathyroid lesions showed hyperplasia histologically in our study. Parathyroid oxyphil cell content is dramatically increased in SHPT [31], even more obviously after treatment with various drugs [32]. Parathyroid lesions are generally larger in PHPT than in SHPT [33–36]. Considering the distinct pathogenesis of SHPT and PHPT, differences in microenvironment, in these patients, may differentially affect MIBI uptake, resulting in lower uptake in SHPT. We found no relationship between age, sex, or biochemical parameters with parathyroid SUVmax which is not consistent with another study [37] probably related to the small number of patients included in our study, or SUVmax adds information independent of baseline measurements but this remains to be investigated.

Parathyroid RI in our study was statistically lower than in thyroid tissue, as illustrated in Fig. 2. This result suggests that 99mTc-MIBI washes out faster from thyroid tissue compared to parathyroid lesions. This likely accounts for the TBR in delayed phase being higher than in the early phase. The lower RI-P than RI-T and higher TBR in delayed phase indicate lower background counts on delayed-phase imaging. Washout of 99mTc-MIBI from parathyroid adenomas in PHPT was also previously shown to be slower than from thyroid tissue [21]. Could single delayed planar imaging be sufficient? In fact, Lavely et al. [16] showed that single imaging had lower sensitivity. In addition, the washout of radiotracer from a parathyroid lesion is not fixed and depends on various factors such as its volume, the number of mitochondria rich oxyphil cells [38], basic clinical characteristics [37] and histologic differences [28]. In contrast to our findings, some studies have reported some adenomas showing rapid release of radiotracer [21, 38] and fast clearance is more common from hyperplastic glands [13, 14]. In addition to these factors concerning washout rate which may account for inconsistencies in the above studies, they may also be related to the use of planar imaging, while we used SPECT/CT. Therefore, delayed-phase imaging alone may miss some parathyroid lesions with fast washout.

Regarding optimal timing of SPECT/CT scanning, some previous studies have compared the diagnostic value of early and delayed 99mTc-MIBI SPECT/CT, with controversial results [14, 17, 39]. Early-phase 99mTc-MIBI SPECT/CT was shown to have an improved sensitivity and a better detection rate in PHPT and SHPT compared to its delayed-phase counterpart [9, 14]. Another study showed early-phase 99mTc-MIBI SPECT/CT gave optimal timing [11]. These studies suggested benefit of early-phase SPECT/CT, mainly due to the rapid washout of MIBI [11]. By contrast, in other studies, delayed-phase SPECT/CT was recommended. Yong-il insisted that delayed 99mTc-MIBI SPECT/CT provided more sensitive information [40]. Kate et al. [15] claimed that the optimum timing of SPECT/CT in PHPT was delayed-phase SPECT/CT due to superior identification compared to early-phase scanning. The application of SUV technology to SPECT/CT provides additional information in parathyroid imaging. It can help identify and differentiate the pathology of parathyroid lesions [30] and assist surgeons in selecting the appropriate parathyroid lesions in SHPT for autotransplant [27]. Suh et al. [30] observed that 99mTc-MIBI uptake of parathyroid hyperplasia lesions in PHPT was similar to that of thyroid tissue in the early phase, but significantly different in the delayed phase, leading to the recommendation to perform SPECT/CT in the delayed phase. Delayed-phase SPECT/CT in SHPT was adopted in later study [27]. Our results are consistent with these later findings; in Fig. 1, we showed that SUVmax in parathyroid lesions and thyroid in SHPT did not differ significantly in the early phase, but was significantly different in the delayed phase. The combined TBR of parathyroid lesions to thyroid in delayed phase was significantly higher than in early phase, and delayed-phase SPECT/CT may be optimal in SHPT. In other studies comparing single-phase and dual-phase SPECT/CT [16, 17], sensitivity and localization accuracy were higher with dual than with single-phase SPECT/CT, but without a statistically significant difference. Performing dual-phase SPECT/CT obviously increased the time of examination. Synthesizing the above, we suggest performing single-phase SPECT/CT, especially delayed phase, in addition to dual-phase planar imaging in SHPT.

We acknowledge some limitations in our study. First, our study is limited by the relatively small cohort of patients. Second, we did not re-analyze by pathology due to the small number of patients. Therefore, larger patient numbers need to be studied in the future.

## Conclusion

Our study demonstrated that SUVmax in parathyroid lesions of SHPT patients was significantly higher than in thyroid tissue on delayed-phase SPECT/CT. The TBR in delayed-phase SPECT/CT was significantly higher than in early-phase SPECT/CT, demonstrating a lower washout of 99mTc-MIBI from parathyroid lesions compared to thyroid gland and a better tumor-to-background ratio in SHPT patients. We conclude that delayed-phase SPECT/CT is required in SHPT because of excellent tumor visualization resulting from high TBR of parathyroid lesions to thyroid tissue.

## Data Availability

The datasets generated and/or analyzed during the current study are available from the corresponding author on reasonable request.

## Abbreviations

SUV: Standardized uptake value
TBR: Target-to-background ratio
SPECT/CT: Single-photon emission computed tomography/computed tomography
SHPT: Secondary hyperparathyroidism
99mTc-MIBI: 99mTc-methoxyisobutylisonitrile
CKD: Chronic kidney disease
sPTX: Surgical parathyroidectomy
Ca: Calcium
P: Phosphorus
ALP: Alkaline phosphatase
PTH: Parathyroid hormone
BUN: Urea nitrogen
Scr: Creatinine
LHER: Low-energy high-resolution
